# Computational prediction of essential genes in an unculturable endosymbiotic bacterium, Wolbachia of Brugia malayi

**DOI:** 10.1186/1471-2180-9-243

**Published:** 2009-11-28

**Authors:** Alexander G Holman, Paul J Davis, Jeremy M Foster, Clotilde KS Carlow, Sanjay Kumar

**Affiliations:** 1 New England Biolabs, 240 County Road, Ipswich, MA 01938-2723, USA

## Abstract

**Background:**

*Wolbachia *(*w*Bm) is an obligate endosymbiotic bacterium of *Brugia malayi*, a parasitic filarial nematode of humans and one of the causative agents of lymphatic filariasis. There is a pressing need for new drugs against filarial parasites, such as *B. malayi*. As *w*Bm is required for *B. malayi *development and fertility, targeting *w*Bm is a promising approach. However, the lifecycle of neither *B. malayi *nor *w*Bm can be maintained *in vitro*. To facilitate selection of potential drug targets we computationally ranked the *w*Bm genome based on confidence that a particular gene is essential for the survival of the bacterium.

**Results:**

*w*Bm protein sequences were aligned using BLAST to the Database of Essential Genes (DEG) version 5.2, a collection of 5,260 experimentally identified essential genes in 15 bacterial strains. A confidence score, the Multiple Hit Score (MHS), was developed to predict each *w*Bm gene's essentiality based on the top alignments to essential genes in each bacterial strain. This method was validated using a jackknife methodology to test the ability to recover known essential genes in a control genome. A second estimation of essentiality, the Gene Conservation Score (GCS), was calculated on the basis of phyletic conservation of genes across *Wolbachia's *parent order *Rickettsiales*. Clusters of orthologous genes were predicted within the 27 currently available complete genomes. Druggability of *w*Bm proteins was predicted by alignment to a database of protein targets of known compounds.

**Conclusion:**

Ranking *w*Bm genes by either MHS or GCS predicts and prioritizes potentially essential genes. Comparison of the MHS to GCS produces quadrants representing four types of predictions: those with high confidence of essentiality by both methods (245 genes), those highly conserved across *Rickettsiales *(299 genes), those similar to distant essential genes (8 genes), and those with low confidence of essentiality (253 genes). These data facilitate selection of *w*Bm genes for entry into drug design pipelines.

## Background

Exponential growth in the amount of available genomic information has produced unprecedented opportunities to computationally predict functional genomics in biologically intractable organisms. One application of these data is facilitation of the rational drug design process. Most high throughput drug discovery techniques screen compounds for biological activity, only determining target and mechanism *post hoc*. An alternative approach, rational drug design, seeks to utilize genomic information to specifically identify and inhibit targets. Often these methods utilize *in silico *sequence analysis to choose a target protein that is important to the survival of the organism and accessible to small molecule drugs. It has been suggested that ideally a target should fulfill four properties: 1--Essentiality to the survival or pathogenesis of the target organism, 2--Druggability, having protein structure characteristics making it amenable to binding small molecule inhibitors, 3--Functional and structural characterization with established assays for screening small molecule inhibition, 4--Distinctness from current drug targets to avoid resistance [1].

These parameters are not strict rules, however. In reality, few if any pathogenic organisms have sufficiently comprehensive functional genomics information to rigorously screen based on these parameters. A large portion of the target discovery process involves weighing compromises in the selection parameters based on the quality of information available. *In silico *drug target prediction relies on various approximations and comparisons to identify genes which fit these parameters. Arguably, the most important parameter to assess is gene essentiality. For a compound to serve as an effective antimicrobial or anthelmintic, binding of its target gene product should kill, or at least severely attenuate the growth of the targeted organism. Knock-out and knock-down studies have been used to assess gene essentiality in a number of diverse model and disease organisms. Recently, many of these studies have been assembled into collection databases [2,3] allowing analyses that examine patterns of essential genes across multiple organisms [4]. In organisms in which a genome wide essentiality survey has not been completed, additional approaches have been used to predict essential genes. If gene essentiality has been determined in a closely related model organism, orthology between genes can predict shared essentiality [5-10]. Alternatively, systems biology approaches examine the global enzymatic and metabolic requirements of the organism. Among these are studies which define a minimal genome for a generic bacterial organism [11-13], or model the total metabolic interactions of the cell [14,15]. For organisms with no functional genomics information in nearby species, methods based purely on gene sequence are being developed, though these provide lower accuracy than functional comparisons [16,17]. Among the purely sequence based methods, gene conservation across taxa is the strongest indicator of gene essentiality [11,16,18,19]. Genes whose protein sequences have been tightly conserved across lineages are assumed to be more likely to be important to the survival of the organism [20]. Each of the essential gene prediction methods described above requires different levels of *a priori *information about the target organism or closely related organisms. As the amount of functional genomics information available decreases, predicting essential genes and drug targets becomes a significantly more difficult task. Here we present the results of our analysis of one such organism having no such functional data, the *Wolbachia *endosymbiont of *Brugia malayi*, (*w*Bm).

*B. malayi *is a parasitic filarial nematode of humans which, along with *Wuchereria bancrofti *and *Onchocerca volvulus*, are the causative agents of lymphatic filariasis and onchocerciasis, more commonly known as elephantiasis and river blindness, respectively. Together, filarial parasites infect approximately 150 million people worldwide with 1.5 billion at risk of infection [21]. Current treatments utilize diethylcarbamazine, benzimidazoles (e.g., albendazole) and avermectins (e.g., ivermectin), however, these treatments are predominately only effective during the larval stages of the parasite [22]. Because the life-span of the adult worm is up to 15 years, long treatment courses are required to effectively eliminate the infection. Additionally, the emergence of drug resistance is becoming increasingly apparent [23,24]. The *α*-proteobacterium *Wolbachia *is an obligate endosymbiont of most filarial nematodes, and in several, including *B. malayi*, is required for worm viability. Clearance of the *Wolbachia *by antibiotics results in worm growth retardation, infertility and killing, while antibiotic treatment of non-*Wolbachia *carrying nematode species has no effect [25,26]. This makes *Wolbachia *an attractive target for control of filarial parasites.

Neither *Wolbachia *nor *B. malayi *have a life-cycle that can be maintained *in vitro*. Because of this, traditional drug discovery by high throughput compound screening is not feasible, nor are the basic gene essentiality experiments which are informative to rational drug design. The genomes of both *B. malayi *and *w*Bm have been sequenced [27,28]; however, only *B. malayi *has a closely related, well characterized model organism, *Caenorhabditis elegans*. Previous work has used *C. elegans *functional genomics data to predict drug targets in *B. malayi *[9]. *Wolbachia*, however, has no close relatives in which functional genomics data is available.

Functional genomics information from a large number of more distantly related bacteria can be used to infer similar information in an intractable species [29,30]. Here we present such an approach, utilizing bioinformatic techniques to rank the likelihood of gene essentiality across the *w*Bm genome, for the purpose of facilitating the selection of potential new drug targets. A combination of approaches were used to predict genes likely to be important to the survival of *w*Bm. First, we used comparative sequence analysis to identify *w*Bm genes with strong protein sequence similarity to experimentally identified essential genes in more distantly related bacteria. Second, in order to identify genes important to the biological niche inhabited by *w*Bm, gene conservation across its parent order, *Rickettsiales *was evaluated. The first approach identifies genes broadly important across bacterial life. The second approach reinforces the genes identified by the first, while additionally identifying genes likely to have importance specifically within *Rickettsiales*. Consideration of these properties during drug target selection can optimize for development of either a more broad spectrum antibiotic, or a more targeted compound, reducing the side effects related to clearing of the natural biotic flora.

## Results

### Predicting essential genes in *w*Bm by protein sequence comparison to essential genes in distantly related bacteria

**Figure 1:**
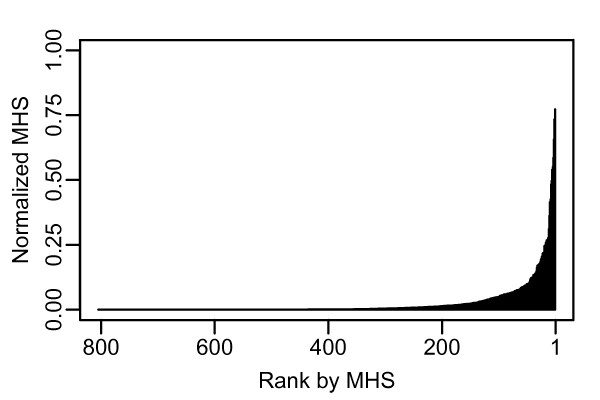
**Distribution of MHS values by rank in *w*Bm**. The X-axis indicates the 805 protein coding genes in the *w*Bm genome, ranked by MHS. The Y-axis shows the value of the MHS for each protein.

**Figure 2:**
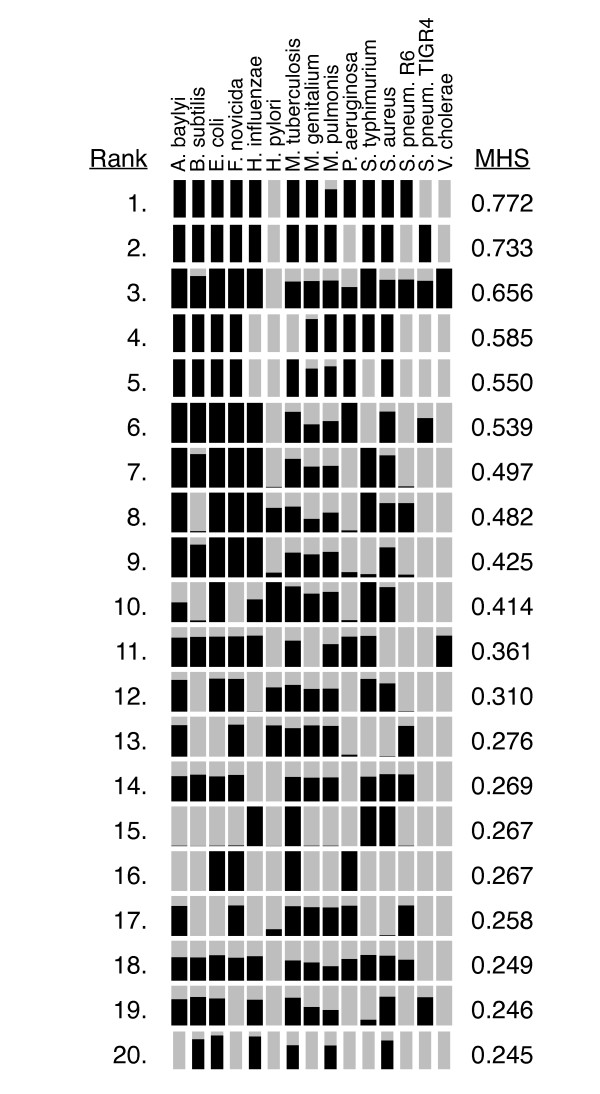
**E-values of the BLAST alignments producing the top 20 MHS**. The black bars indicate the e-value of the best alignment to each organism within DEG. The y-axis is a linear scale of the negative log_10 _of the e-value, ranging from 1 to a maximal alignment of 200. The x-axis bins correspond to the 15 organisms contained within DEG.

While *w*Bm is not amenable to experimental gene essentiality analysis, knockout and knockdown studies in multiple other bacterial species can serve as a proxy. The results of a number of these analyses are compiled in a publicly available resource called the Database of Essential Genes (DEG). This database contains 5,260 genes from 15 different bacterial strains [3] (Table 1). In most cases, the genes within DEG were identified by large scale knock-out or knock-down screens performed under rich media conditions. Rich media conditions are thought to approximate the growth environment of intracellular bacteria [16]. This makes the collection of genes within DEG a useful model for the gene requirements of *w*Bm. DEG contains a binary description of gene essentiality. Genes included in DEG are considered essential to the organism, while genes omitted are considered dispensable, within the specific conditions of the experiments used. In order to computationally predict essential genes, we used BLAST to compare the protein sequences of all protein-coding *w*Bm genes to the genes contained within DEG. The most straightforward method to evaluate the results from the BLAST analysis is to examine the e-value of the best BLAST hit between a *w*Bm gene and DEG. However, because DEG consists of information on essential genes in multiple bacterial organisms, we wished to evaluate the BLAST results in a manner which accounts for the statistical significance of hits to multiple DEG organisms. A *w*Bm gene with a significant BLAST hit to an essential gene in a single DEG organism represents a quite different result than a *w*Bm gene with significant BLAST hits to essential genes in multiple DEG organisms. While a single alignment to a DEG gene implies similar function and likely shared essentiality, alignments to DEG genes within multiple organisms suggests membership in a class of essential genes conserved across species and increases our confidence in predicting that a given *w*Bm gene is essential. A ranking metric, termed the multiple-hit score (MHS), was developed to evaluate the BLAST results in this context. This metric produced a score for each *w*Bm gene. A gene with high-scoring BLAST hits to each organism within DEG received a high MHS score. In its basic form, the MHS for a *w*Bm gene was calculated by averaging the top BLAST alignment against each DEG organism divided by the smallest e-value able to be returned by BLAST, 1 × 10^-200 ^in this case. The scale of e-values generated by BLAST are dependent on the size of the database searched [31]. Preliminary analysis indicated that when searching against the DEG database, e-values less significant than 1 × 10^-25 ^were predominately partial alignments (data not shown). To reduce the effect of these lower significance alignments, which appeared to be domain alignments instead of full length gene alignments, all e-values were scaled by their square before averaging. The resulting score could range between 0 and 1, with 1 being alignments with an e-value of 1 × 10^-200 ^to all organisms within DEG. Figure 1 is a graph of the MHS scores for the full *w*Bm genome, ordered by MHS score [see Additional file 1]. This graph reveals several properties of the *w*Bm MHS distribution. There is a sharp peak containing fewer than 10 genes which have very good alignments to nearly all DEG organisms. This tapers to a shoulder containing, first, genes with high quality alignments to several DEG organisms, then later, mostly genes with lower quality alignments to multiple DEG organisms. The distribution of actual alignments for the top 20 genes is shown in Figure 2. Because the MHS indicates our confidence that a specific gene is essential, the optimal usage of this ranking is to begin manually examining from the highest ranked genes, progressing through genes with a lower confidence of essentiality. Based on the shape of the MHS curve and examination of the individual alignments, a conservative MHS threshold of 7.3 × 10^-3 ^was chosen. At this threshold, we see alignments to 7 of the 15 taxa in DEG with e-values of 1 × 10^-25^. This threshold predicts that 250 out of 805 genes have reasonable confidence of essentiality. This should not, however, be mistaken as a prediction that two-thirds of the genome is non-essential. As an obligate endosymbiont of the nematode *B. malayi*, *w*Bm has undergone significant genome shrinkage compared to other bacteria, thus a large percentage of its genome is expected to be essential [28]. Instead, the MHS result predicts that roughly one-quarter of the *w*Bm genes are involved in basic bacterial processes important for growth across a diversity of species. Identification of a supplementary set of genes consisting of genes likely to be important specifically to members of the order *Rickettsiales *was accomplished in the second phase of our analysis.

**Table 1:** DEG Members

Organism Name	Taxon ID	Ess. Genes	Refseq Gene Count	% Ess.
*Acinetobacter baylyi ADP1*^*γ*^	202950	499	3325	15%
*Bacillus subtilis 168*^B^	224308	271	4105	7%
*Escherichia coli MG1655*^*γ*^	511145	712	4132	17%
*Francisella novicida U112*^*γ*^	401614	392	1719	23%
*Haemophilus influenzae Rd KW20*^*γ*^	71421	642	1657	39%
*Helicobacter pylori 26695*^ϵ^	85962	323	1576	20%
*Mycobacterium tuberculosis H37Rv*^A^	83332	614	3989	15%
*Mycoplasma genitalium G37*^M^	243273	381	477	80%
*Mycoplasma pulmonis UAB CTIP*^M^	272635	310	782	40%
*Pseudomonas aeruginosa UCBPP-PA14*^*γ*^	208963	335	5892	6%
*Salmonella typhimurium LT2*^*γ*^	99287	230	4527	5%
*Staphylococcus aureus N315*^B^	158879	302	2619	12%
*Streptococcus pneumoniae R6*^B^	171101	133	2043	12%
*Streptococcus pneumoniae TIGR4*^B^	170187	111	2105	12%
*Vibrio cholerae*^*γ*^	243277	5	3835	0%

(*γ*): *γ*-proteobacteria, (B): bacilli, (ϵ): ϵ-proteobacteria, (A): actinobacteria, (M): mollicutes.

### Evaluation and validation of the MHS ranked *w*Bm gene list

**Figure 3:**
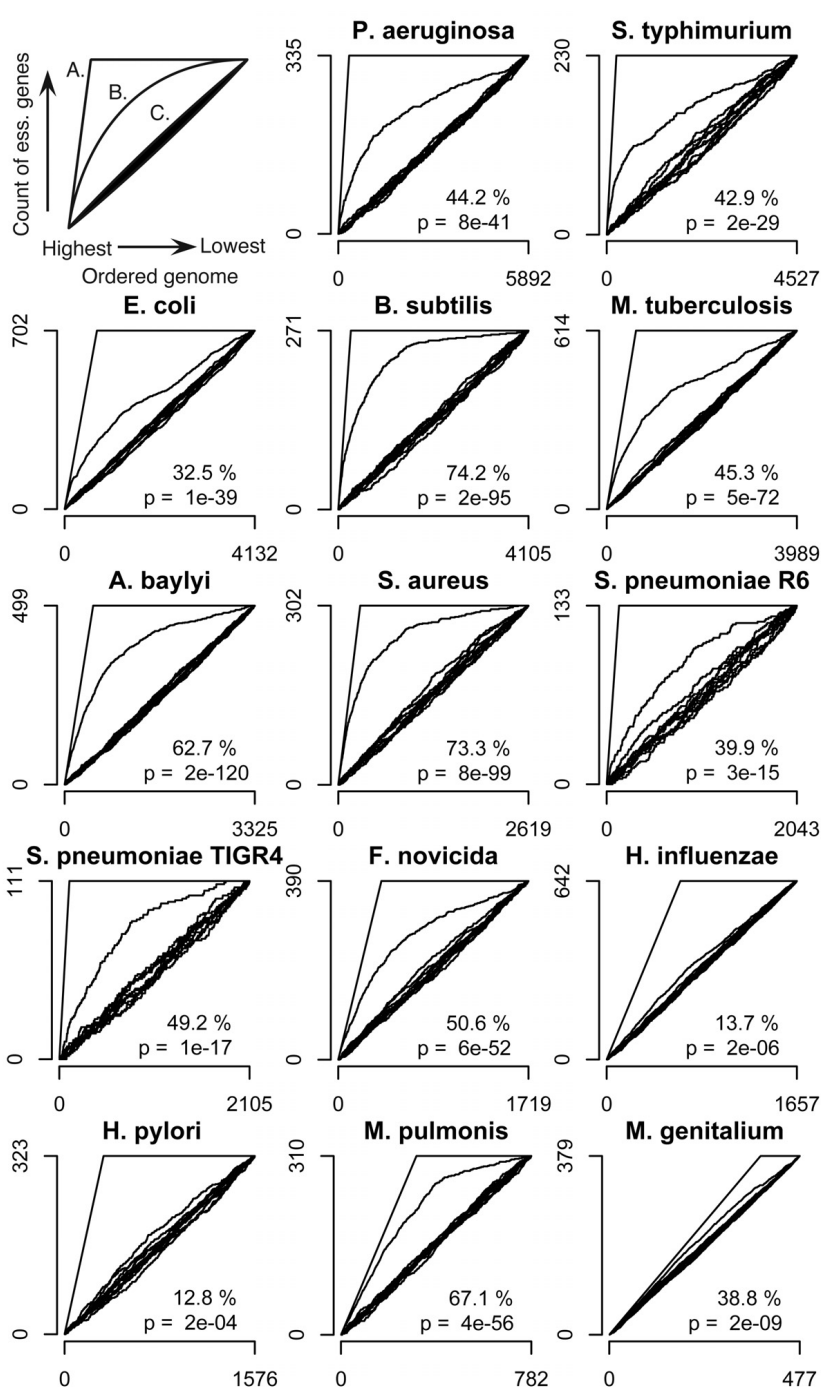
**Essential gene prediction by MHS was validated through a jackknife methodology**. For each organism within DEG, the ability of the MHS to place experimentally validated essential genes at the top of a ranked genome was evaluated. All graphs correspond to the schematic found in the upper left. The X-axis represents the ranked genome of the organism, ranked from left to right as strongest to weakest prediction of essentiality. The Y-axis is the cumulative count of essential genes encountered moving left to right through the ranked genome. Line A is the ideal sorting, in which all essential genes are placed at the top of the ranking. Line B is the sorting by MHS. Lines C are 10 random assortments of the genome. Percent sorting achieved by MHS and the p-value for the difference between the MHS score ranking B and 1000 random assortments such as in C are shown in the lower right. Graphs are ordered by descending genome size of the organism. *E. coli*, *F. novicida*, and *M. genitalium *show 10, 2 and 2 fewer total essential genes, respectively, than shown in Table 1 because the corresponding DEG genes are not able to be resolved to genomic genes and are omitted from the jackknife analysis.

The annotations of the top 20 *w*Bm genes ranked by MHS can be used to qualitatively assess our ranking metric (Table 2). Many of the top-20 genes fall into the classes of genes targeted by current antibiotics and are annotated in categories likely essential for bacterial growth. The gyrase and topoisomerase family, targeted by quinolones [32], is heavily represented. The DNA-directed RNA polymerase RpoB is the target of rifampin [33], and the tRNA synthetases are targets of several recently developed compounds [34-36]. In addition to qualitatively examining our ranking method, we wanted to quantitatively assess its ability to place essential genes at the top of the ranked list. However, quantitatively validating the ranking of the *w*Bm genome is stymied by the lack of an effective positive control set. To address this we developed a jackknifing methodology which is able to utilize the organisms within DEG as a positive control set with which to validate the ranking methods. The Refseq sets of predicted proteins for organisms included in DEG were acquired from NCBI. Each organism's protein sequences were individually analyzed by comparison to a version of DEG filtered to remove sequences from just that organism, then ordered by MHS. Because essential genes in these organisms have already been experimentally identified, it is possible to assess our ranking methods by their ability to prioritize these genes. In order to quantitate the ranking, each genome was ordered by highest to lowest prediction of essentiality and the cumulative sum of the number of positive control DEG genes was plotted. The area under the curve (AUC) for the experimental ranking was compared to that of an ideal ranking which artificially placed all DEG genes at the beginning of the list, and 1000 replicates of a randomized assortment (Figure 3). The shape of the ideal and sorted curves varies with the percentage of DEG genes within each organism. The important component to examine is the shape of the experimental sorting curve compared to the randomized assortment and the ideal ranking. For each organism a p-value was calculated, comparing the experimental sorting with the randomly assorted population. Additionally, the percentage sorting was calculated by scaling the area under the curve for the experimental sorting to between 100% for the area under the curve in the ideal ranking, and 0% for the AUC for the diagonal line representing random assortment. Qualitatively, for most organisms our methods performed relatively well in recovering DEG genes. In nearly all organisms the sorted curve appears well differentiated from the randomized sorting and in some cases begins to approach the ideal case. For all organisms the experimental sorting was statistically different from random assortment. *B. subtilis*, *S. aureus*, and *M. pulmonis *are examples of organisms with large, medium and small genomes which were especially well sorted by MHS, with 74.2%, 73.3% and 67.1% sorting respectively. On the other hand, *H. influenzae *and *H. pylori *and to a lesser extent *E. coli *performed quite poorly in this validation with 13.7% 12.8% and 32.5% sorting respectively. Further consideration of these outliers can be found in the discussion. Overall, the results from the jackknife analysis indicate that the MHS based ranking effectively predicts essential genes and prioritizes them within the top of the ranked genome.

**Table 2:** Top 20 *w*Bm genes ranked by MHS. Annotations taken from the Refseq release of the *w*Bm proteome.

Rank	MHS	GI	Annotation
1	0.772	58584904	DNA-directed RNA polymerase: RpoB/RpoC
2	0.733	58584602	Translation elongation factor GT-Pase: FusA
3	0.656	58585021	DNA gyrase, topoisomerase II, B sub-unit: GyrB
4	0.585	58584662	DNA gyrase subunit A
5	0.550	58584524	Translocase
6	0.539	58584756	DNA polymerase III alpha subunit
7	0.497	58584618	Alanyl-tRNA synthetase
8	0.482	58584729	Threonyl-tRNA synthetase
9	0.425	58584862	Leucyl-tRNA synthetase
10	0.414	58584752	Molecular chaperone: DnaK
11	0.361	58584429	CTP synthetase
12	0.310	58584410	ATP-dependent Zn protease: HflB
13	0.276	58584946	ATP synthase subunit B
14	0.269	58584379	Enolase
15	0.267	58584441	ATP-binding subunit of Clp protease and DnaK/DnaJ chaperones
16	0.267	58584652	2-oxoglutarate dehydrogenase complex, E1 component
17	0.258	58584572	ATP synthase subunit A
18	0.249	58584805	NAD-dependent DNA ligase: Lig
19	0.246	58584298	Topoisomerase IA: TopA
20	0.245	58584921	Transketolase

### Prediction of essential genes in *w*Bm by gene conservation across the order Rickettsiales

While we are confident in the predictions of gene essentiality by MHS, those predictions only identify genes common to the reference set of bacteria in DEG. As there are no *α*-proteobacteria in DEG, genes uniquely essential to *w*Bm might be missed by MHS analysis. We wished to perform a complementary analysis to predict additional genes important specifically to *w*Bm and closely related organisms. *w*Bm is a highly specialized obligate endosymbiont with a reduced genome [28]. While it seems reasonable that roughly 250 out of 805 *w*Bm genes are essential across bacteria in general, it is likely that there is an additional set of genes essential specifically for the environmental niche inhabited by *w*Bm. In order to predict this second set of genes, and reinforce the MHS based essential gene predictions, we identified genes with highly conserved orthologs across *Wolbachia's *parent order, *Rickettsiales*.

There are 27 complete genomes available within *Rickettsiales*. These include, 4 *Wolbachia*, including *w*Bm, 3 genomes from the genus *Anaplasma*, 5 *Ehrlichia*, 11 *Rickettsia*, 1 *Neorickettsia*, 2 *Orientia*, and 1 *Pelagibacter *(Table 3). Of these genomes, all but *Pelagibacter *are obligate endosymbionts residing either in vacuoles or within the host cell cytoplasm. Of the endosymbionts, all but *Wolbachia *replicate within vertebrate hosts with most transmitted via an invertebrate vector. *Wolbachia*, on the other hand infects a diverse spectrum of arthropod hosts as well as filarial nematodes, many of which are themselves vertebrate parasites [37].

**Table 3:** Genomes available within the order Rickettsiales

Genus species Strain	Taxon ID
*Anaplasma marginale St Maries*	234826
*Anaplasma phagocytophilum HZ*	212042
*Anaplasma marginale Florida*	320483
*Candidatus Pelagibacter ubique HTCC1062*	335992
*Ehrlichia canis Jake*	269484
*Ehrlichia chaffeensis Arkansas*	205920
*Ehrlichia ruminantium Gardel*	302409
*Ehrlichia ruminantium Welgevonden UPSA*	254945
*Ehrlichia ruminantium Welgevonden CIRAD*	254945
*Orientia tsutsugamushi Boryong*	357244
*Orientia tsutsugamushi Ikeda*	334380
*Neorickettsia sennetsu Miyayama*	222891
*Rickettsia akari Hartford*	293614
*Rickettsia bellii OSU 85-389*	391896
*Rickettsia bellii RML369-C*	336407
*Rickettsia canadensis McKiel*	293613
*Rickettsia conorii Malish 7*	272944
*Rickettsia felis URRWXCal2*	315456
*Rickettsia massiliae MTU5*	416276
*Rickettsia prowazekii Madrid E*	272947
*Rickettsia rickettsii Iowa*	452659
*Rickettsia rickettsii Sheila Smith*	392021
*Rickettsia typhi wilmington*	257363
*Wolbachia Drosophila melanogaster*	163164
*Wolbachia Drosophila simulans*	66084
*Wolbachia Culex quinquefasciatus*	570417
*Wolbachia Brugia malayi TRS*	292805

**Figure 4:**
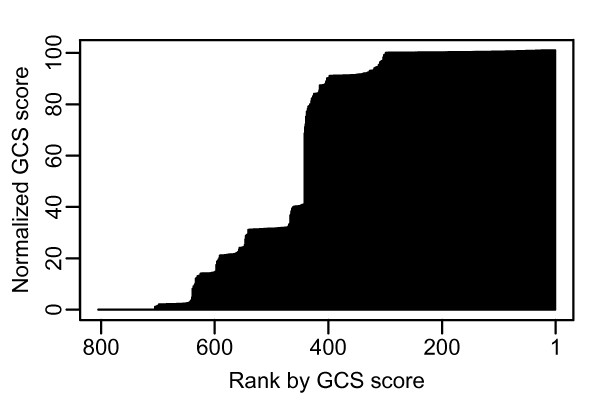
**Distribution of GCS in *w*Bm**. The X-axis indicates the 805 protein coding genes in the *w*Bm genome, ranked by GCS. The Y-axis shows the value of the GCS for each protein.

**Figure 5:**
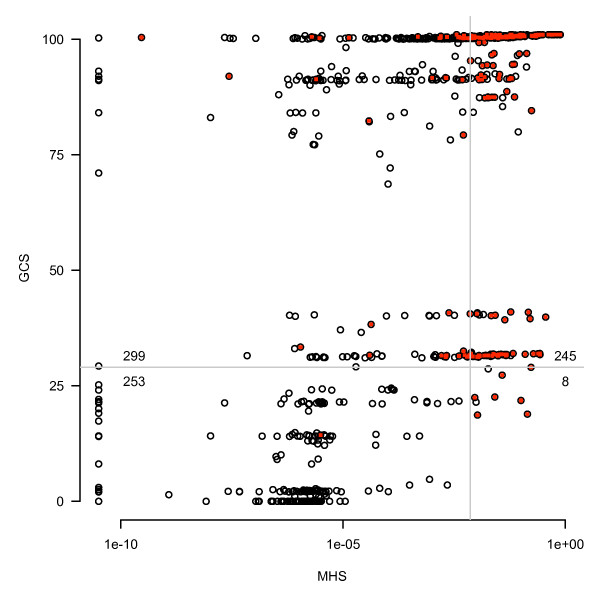
**Comparison of the prediction of *w*Bm gene essentiality by MHS and GCS**. The X-axis shows normalized MHS on a log scale, while the Y-axis shows GCS. Grey lines indicate empirically determined thresholds for confidence in prediction of essentiality and are set at 7.3 × 10^-3 ^for the MHS and 29 for the GCS. Therefore, the upper right quadrant contains genes with high confidence by both metrics. The upper left quadrant contains genes identified only by GCS, while the bottom right quadrant contains genes identified only by MHS. The numbers adjacent to the quadrant lines indicate gene counts in each quadrant. Red dots indicate Wolbachia genes which have significant protein sequence similarity to the targets of approved drugs and are predicted to be druggable.

Refseq protein sequences from the 27 available genomes (as of April 1, 2009) were retrieved from NCBI. The OrthoMCL package was used to predict clusters of orthologs among the genomes [38]. To gauge the extent of taxonomic diversity within each orthologous gene cluster, we initially tallied the number of taxa represented in the cluster. However, this measure inflated the phylogenetic diversity for groups containing multiple highly related taxa. To compensate, a minimum spanning tree (MST) was constructed using distances derived from aligned 16S rRNA gene sequences as edge weights between taxonomic nodes. A score for the MST was calculated by summing the distances between the connected taxonomic nodes. The MST was used to minimize the contributions from closely related taxa, while reflecting the overall taxonomic diversity. The MST distances for each cluster were incorporated into a metric we termed the gene conservation score (GCS), which represents both the extent of gene conservation across species, as well as the quality of that conservation. The integer portion of the GCS, from 0 to 100, is derived from the MST distances within an orthologous gene cluster. The decimal portion of the score represents the quality of alignments between the *w*Bm gene and the other cluster members. Thus, within a group of clusters with the same MST, *w*Bm genes are individually ranked based on the quality of their BLAST alignment to other genes within the cluster (see Materials and Methods). The distribution of GCS scores for the *w*Bm genome is shown in Figure 4 [see also Additional file 1]. Approximately 300 *w*Bm genes cluster with orthologs in all or nearly all *Rickettsia *members in the analysis and have a GCS of approximately 100. The next large group consists of 60 *w*Bm genes that have a GCS of approximately 91 and orthologs in all members except for *Pelagibacter ubique*, the only free-living organism in the group. A third group of 60 genes has a GCS of approximately 29, and corresponds to clusters lacking orthologs to *Orientia *and most of the *Rickettsia *species. When picking an empirical threshold for prediction of gene essentiality we chose a GCS of 29 or higher, which includes the three groups described above and contains 544 genes. Though the third group of 60 genes has lost orthologs to most of the *Rickettsia*, it retains orthologs in the *Anaplasma*, *Ehrlichia*, *Neorickettsia *and the other *Wolbachiae*. As is illustrated by the distribution along the y-axis of Figure 5, however, there is a large break between groups with a GCS of 91 and 29, and a more conservative estimate could place a threshold significantly higher. From a practical standpoint, however, because the GCS value represents a prediction of the importance of a specific gene, a more useful approach is to sort the genome by GCS rather than picking a threshold. Manually assessing from the top of the ranking allows the identification of highly conserved genes which can be searched for favorable secondary protein properties; in our case, properties useful for entry into the rational drug design pipeline.

Examination of the top 20 genes in the *w*Bm genome ranked by GCS (Table 4) reveals important differences compared to ranking by MHS (Table 2). Many of the same genes or classes of genes which were ranked highly by MHS are also identified by GCS. RNA polymerase RpoB/C, topoisomerase, gyrase, and several tRNA synthetases all rank highly by both methods. However, several interesting genes not identified by MHS are placed at the top of the GCS ranking. For example, pyruvate phosphate dikinase, PPDK, has previously been identified by pathway analysis as a potential drug target [39]. By MHS, PPDK was ranked at position 309; GCS ranking placed it at position 3.

**Table 4:** Top 20 *w*Bm genes ranked by GCS. Annotations taken from the Refseq release of the *w*Bm proteome.

Rank	GCS	GI	Annotation
1	101	58584652	2-oxoglutarate dehydrogenase complex, E1 component
2	101	58584298	Topoisomerase IA: TopA
3	101	58584469	Pyruvate phosphate dikinase
4	101	58584904	DNA-directed RNA polymerase: RpoB/RpoC
5	101	58584952	Ribonucleotide-diphosphate reductase alpha subunit
6	101	58584808	ATP-dependent Lon protease
7	101	58584662	DNA gyrase subunit A
8	101	58584705	Succinate dehydrogenase
9	101	58584602	Translation elongation factor, GT-Pase: FusA
10	101	58584729	Threonyl-tRNA synthetase
11	101	58584633	NADH dehydrogenase gamma sub-unit
12	101	58584752	Molecular chaperone: DnaK
13	101	58584862	Leucyl-tRNA synthetase
14	101	58584524	Translocase
15	100.994	58585021	DNA gyrase, topoisomerase II, B sub-unit: GyrB
16	100.989	58584924	GTP-binding protein: LepA
17	100.987	58584410	ATP-dependent Zn protease: HflB
18	100.986	58584731	NADH:ubiquinone oxidoreductase, NADH-binding, chain F
19	100.974	58584620	Isoleucyl-tRNA synthetase
20	100.974	58584756	DNA polymerase III alpha subunit

### Plotting MHS versus GCS demonstrates the identification of complementary sets of essential genes

The two methods of essential gene prediction used in this study identified complementary partially overlapping sets of *w*Bm genes. Identification of a gene by both methods bolsters confidence in a prediction of essentiality. Genes uniquely identified by an individual method may represent, for MHS, genes essential to general bacterial processes; and for GCS, genes specifically important to the *Rickettsiales *order. To assess the distribution of essentiality prediction by both methods, the MHS and GCS for each *w*Bm gene was graphed as a scatter plot (Figure 5). Lines indicating the empirically determined thresholds for the prediction of essentiality by each method produce four quadrants showing the classes of predicted essential genes. The upper-right quadrant contains 245 genes predicted essential by both methods. The upper-left quadrant contains 299 genes which are not similar to essential genes in more distantly related bacteria, but are still highly conserved across *Rickettsiales*. These genes represent a promising class of drug targets which are likely to be more specific to *w*Bm. That there are only 8 outliers in the lower-right quadrant demonstrates that most genes which are predicted to be essential in multiple diverse bacteria are also highly conserved across *Rickettsiales*, as expected. Combined, we predict that 552 of 805 *w*Bm genes--roughly 69%--have a high likelihood of being essential.

### The ranked *w*Bm genome as a tool for drug development

Our ranking of the *w*Bm genome by predicted gene essentiality is designed as a tool to facilitate the manual exploration of viable new drug targets against the bacterium. Order within the list at a resolution of one or two positions is relatively uninformative; nearby rankings represent similar confidence in the prediction of gene essentiality. However, the quartile or decile in which a gene is placed strongly influences our confidence in its essentiality. In addition to predicting essential genes, each *w*Bm gene can be further annotated to include protein or functional information useful in drug target prioritization, including similarity to human proteins, hydropathy predictions, or protein localization predictions. A similar strategy for prioritizing targets was used for *B. malayi *[9] and *Mycobacterium tuberculosis *[40]. One such annotation we chose to include is the potential for a protein to bind typical small molecule drugs, termed its druggability. There exist several purely sequence based methods of predicting druggability based on the identification of domains favorable to small molecule binding [41,42]. We also decided to take a more direct approach and identify *w*Bm proteins with high sequence similarity to the targets of existing small molecule drugs and compounds. This allows us to not only identify proteins containing domains favorably structured to bind small molecules, but also proteins which are likely to have the localization and cellular kinetics important for a viable drug target.

We utilized the DrugBank database which is a comprehensive set of nearly 4,800 FDA-approved small molecule drugs, nutraceuticals and experimental compounds [43]. This database includes chemical, pharmacology, and mechanistic information for each compound, as well as protein target and pathway information for a large percentage of the entries. After downloading a local copy of the database, we used BLAST to align the *w*Bm proteins to the list of drug targeted proteins from DrugBank, filtering for e-values more significant than 1 × 10^-25^. This method identified 198 *w*Bm proteins highly similar to the binding partners of FDA approved drugs, experimental small molecule compounds, or nutraceutical compounds. In Figure 5 druggability is indicated by coloring predicted druggable *w*Bm genes red. The prediction of druggability seems to correlate well with our predictions of potential drug targets by essentiality and gene conservation. In combination with essentiality predictions, the prediction of druggability can be used as a secondary screening criteria to identify genes for entry into the rational drug design pipeline.

## Discussion

The overall goal of this work is to produce a result that can facilitate the selection of genes as drug target candidates. Sorting the full genome by prediction of essentiality then manually evaluating secondary protein properties attempts to avoid the issues related to developing a nuanced automated system capable of filtering down to a short list of candidate drug targets while still prioritizing the listing for high quality potential targets.

**Figure 6:**
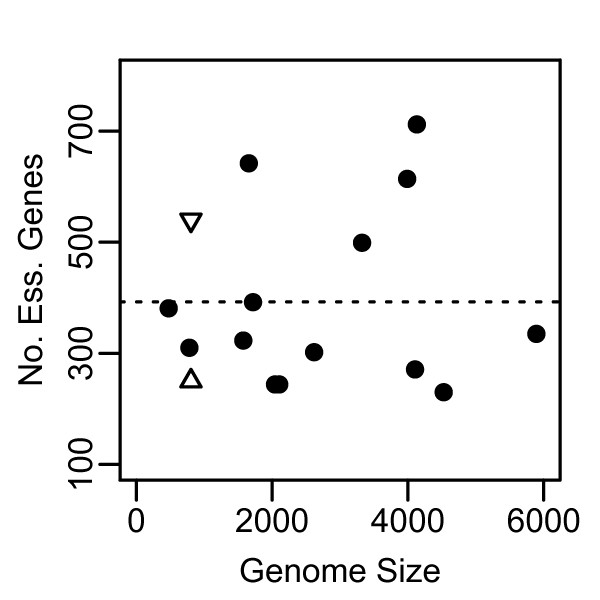
**Number of essential genes versus total number of Refseq genes**. •-DEG organisms (*V. cholerae *omitted as an outlier). △-*w*Bm essential gene prediction by MHS. ▽-*w*Bm essential gene prediction by GCS score.

MHS predicted a slightly smaller number of essential genes than experimentally found in the individual genome surveys comprising DEG. In contrast, GCS predicted a slightly larger set (Figure 6). Because most of the entries within DEG represent genome wide surveys for essential genes we can compare the number of genes identified by our analysis to the number of essential genes in each DEG organism. *Vibrio cholerae *was removed as an outlier because it consists of 5 genes in DEG and does not represent a comprehensive genome survey. By MHS our analysis predicted approximately 250 genes or approximately 30% of the *w*Bm genome as having reasonable confidence of essentiality. The raw number of predicted essential genes is lower than that for most of the DEG organisms, and under the mean for DEG of 392 genes. *Mycoplasma genitalium *and *Mycoplasma pulmonis*, which are also intracellular bacteria with genome sizes similar to *w*Bm, have 381 and 310 genes within DEG, respectively. The relatively similar number of essential genes identified across DEG organisms suggests that these data are describing a common set of genes across a shared set of important pathways. It appears that we are able to predict a quite significant portion of these in *w*Bm through the MHS, though it does appear that MHS alone may not be identifying the complete set. By GCS we identified 544 *w*Bm genes as important within *Rickettsiales*, comprising approximately 69% of the *w*Bm genome. This is greater than the *Mycoplasmas *and most other DEG organisms, but still less than *Haemophilus influenzae *(642), *M. tuberculosis *(614), or *Escherichia coli *(712) (Table 1). Overall, it appears that for prediction of essential genes both MHS and GCS score are effective. MHS is likely an incomplete survey. GCS prediction appears to identify a more complete set, encompassing all but 8 of the genes identified by MHS. However, the additional genes identified by GCS also probably include a number of genes that, while important, are not strictly essential. It is possible to overestimate the set of essential genes predicted by GCS as a result of using closely related organisms. Although we note that in the case of *Rickettsiales*, these organisms are in the process of reducing their genomes, adding significance to retained genes. Within the goals of this research, predicting essential genes as potential drug targets, our methods provide sufficient sensitivity and specificity as long as these caveats are recognized.

On the basis of the jackknife validation, MHS performs poorly on several organisms. *M. genitalium *represents a unique case; nearly 80% of its genes are essential. There is little difference between the AUC for the ideal sorting, the MHS sorting, and the random assortment. Even so, MHS produced a 38.8% sorting, with a p-value of 2 × 10^-9 ^compared to random. It is unclear why *H. influenzae *and *H. pylori *and to a lesser extent *E. coli *performed poorly. This result suggests that these organisms may contain species specific essential genes. For *H. pylori *the authors of the initial essentiality screen note a surprising lack of overlap with the essential gene sets from other organisms [44]. As the number of essential genes in *H. pylori *is in the same range as most of the other organisms in DEG, this could suggest an alternative set of essential genes. In the case of *E. coli*, we note that the number of essential genes is nearly double the average for the other DEG organisms, which likely reflects its status as one of the most well-studied bacteria. This larger set may confound the *E. coli *jackknifing validation. Somewhat paradoxically, these features may be beneficial for this analysis. The outlier organisms may incorporate more diversity in our reference set of essential genes, increasing the likelihood of identification of diverse essential genes within *w*Bm. This does come with the trade-off of increasing the false positive rate, however, this is mitigated by two factors. First, the design of the MHS assigns more confidence to genes conserved across multiple organisms, moving well supported essential gene predictions towards the top. Second, the pipeline for the rational drug design process utilizes the predictions of essential *w*Bm genes to inform a manual selection of drug targets. A moderate false positive rate can be screened out based on manual analysis and pathway information. As an additional experiment, it could be informative to examine non-DEG genes predicted as essential in the jackknifing validation to identify essential genes missed by the knockout experiments. A gene conserved nearly universally across DEG but missing in a small number of organisms may be useful to investigate under alternative experimental conditions.

Genes identified by MHS are predicted to belong to a set of genes which are essential and broadly conserved across bacterial life. This set includes many targets of modern broad-spectrum antibiotics. A compound targeting genes from this class is more likely to produce antibiotics effective across a broad range of bacterial species. Though gene orthology does not specifically indicate drug cross-reactivity, the distribution of the targeted gene should be considered. While developing a novel broad-spectrum antibiotic would be advantageous, for this specific application such a compound may also come with negative side-effects. Ideally, a mass drug administration protocol against *B. malayi *would consist of a small number of high-dose antibiotic treatments. In this format, broad-spectrum antibiotics carry the risk of significant side-effects due to targeting mutualistic bacterial flora.

An alternative approach which attempts to avoid the issues surrounding broad-spectrum antibiotics is to select targets from the group of genes identified only by the GCS. These genes are highly conserved throughout the order *Rickettsiales *but have little similarity to essential genes in other bacteria. While it is quite possible that these *w*Bm genes have orthologs throughout the bacterial kingdom, the experimental data available in DEG suggests that they would not be essential for the growth of bacteria in general. Druggability was predicted by identifying *w*Bm proteins with sequence similarity to the targets of small molecule drugs. However, an intriguing secondary application exists. Comparison of *w*Bm proteins to drug targeted proteins additionally produces a list of approved drug and drug-like compounds which bind proteins of similar sequences to *w*Bm proteins. Protein sequence similarity does not guarantee identical structures or binding pockets, thus it is unlikely that a single turn-key compound will be identified through target similarity. However, it seems reasonable that careful filtering of this set could reveal a panel of potential binding compounds primed for optimization and derivatization using traditional medicinal chemistry. This opens the interesting possibility of applying bioinformatic analysis to bypass a portion of the arduous *de novo *drug development pipeline.

## Conclusion

Through this analysis we were able to predict genes important for the survival of a biologically intractable organism using two complementary bioinformatic techniques. These predictions can then be used as a tool to facilitate the selection of genes to enter into the drug development process against this organism. Comparison of the two predictions revealed that different but overlapping sets of genes were predicted, stemming from the approaches applied. By MHS, 253 genes were predicted as having a high likelihood of being essential. All but 8 of those genes were also identified by the second method, GCS. An additional 299 genes were also identified by GCS alone as highly conserved in *Wolbachia's *parent order *Rickettsiales*. Overall, 552 *w*Bm genes, approximately 69% of the genome, were identified as having a high confidence in a prediction of essentiality. The overlapping and uniquely identified sets of genes can facilitate alternative approaches for drug target selection.

## Methods

### BLAST against DEG

The 805 Refseq protein sequences for the *Wolbachia *endosymbiont of *B. malayi *strain TRS were downloaded from the NCBI ftp site ftp://ftp.ncbi.nlm.nih.gov/genomes/Bacteria. The Database of Essential Genes (DEG) version 5.2 was provided by Dr. Ren Zhang at the Centre of BioInformatics, Tianjin University. The standalone release of the BLAST sequence alignment program version 2.2.19 was obtained from the NCBI BLAST website [45]. Using default parameters, blastp was used to align the *w*Bm protein sequences against the protein sequences contained in DEG. To produce the multi-hit score, the negative *log*_10 _of the e-values of the highest scoring alignments to each of the DEG organisms were normalized between 0 and 1, squared, then averaged for all DEG organisms. E-values greater than 1 were truncated at 1.

Where *N *= the number of DEG organisms and 1 × 10^-200 ^is the smallest e-value reported by BLAST.

### Jackknife Analysis

Complete Refseq protein sequences for the 15 organisms contained within DEG were downloaded from the NCBI Refseq ftp site ftp://ftp.ncbi.nlm.nih.gov/genomes/Bacteria. For each organism, a filtered version of DEG was prepared, removing just the proteins from that organism. The full protein complement of that organism was then subjected to MHS analysis using the filtered version of DEG, and ranked based on MHS. Moving through the ranked genome from highest prediction of essentiality to lowest, the cumulative sum of DEG genes encountered was calculated. The area under the curve (AUC) of the cumulative sum describes the effectiveness of the ranking. The upper bound of the AUC is defined by an ideal sorting which places all DEG genes at the top of the list. The mean and standard deviation of the AUC for the null hypothesis of no sorting was determined by randomly permuting the genome sorting 1000 times. The AUCs for the random assortments was assumed to represent a normal distribution with the observed mean and standard deviation. The p-value of the MHS sorting versus the null hypothesis was calculated using the probability density for a normal distribution. For the calculation of percent sorting, the AUC for the unsorted diagonal was one-half of the total area of the graph, calculated as the total number genes in the genome multiplied by the number of DEG genes, divided by two.

### Gene Conservation Across Rickettsiales

Refseq protein sequences were downloaded from the NCBI Refseq ftp site for the 27 sequenced organisms in the order *Rickettsiales *(Table 3). The standalone version 1.4 of the OrthoMCL ortholog prediction program was downloaded http://www.orthomcl.org/common/downloads/software/[38]. OrthoMCL was used with default settings and an inflation value of 1.5 to predict orthologs among the protein sequences of the 27 genomes. Briefly, OrthoMCL begins by using an all-versus-all BLAST search to identify reciprocal best BLAST hits among the genomes as putative orthologs, and reciprocal best BLAST hits within genomes as putative in-paralogs. These interconnections are used to form a similarity graph that is used by the MCL clustering algorithm to break mega-clusters into suitable sub-clusters of orthologs [46].

For each cluster of orthologous genes the minimum spanning tree (MST) distance was calculated based on the phylogenetic distances among the member genomes. The 16S rRNA gene was extracted from each of the complete genome sequences used in this study (Table 5). A multiple sequence alignment of the 16S genes was generated with Muscle v3.41 [47] using default values for maximum iterations and maximum time. A distance matrix was generated from the aligned sequences with the dnadist program from the Phylip suite v3.68 using the Kimura 2-parameter distance model. For each orthologous cluster, we extracted the taxon IDs of the taxa included in the cluster. Using the calculated distances between taxa based on aligned 16S sequences as edge weights between the taxon nodes, a minimum spanning tree (MST) was generated using Prim's algorithm [48]. Each MST was scored based on the sum of edge weights included in the tree.

**Table 5:** 16S rRNA gene sequence sources

Refseq ID	Taxon	Coordinates	Species name
NC_012026.1	320483	246283-247795	*Anaplasma marginale str. Florida*, complete genome
NC_004842.2	234826	247468-248989	*Anaplasma marginale str. St. Maries*
NC_007797.1	212042	1057470-1058902	*Anaplasma phagocytophilum HZ*
NC_007205.1	335992	511358-512831	*Candidatus Pelagibacter ubique HTCC1062*
NC_007354.1	269484	285955-287439	*Ehrlichia canis str. Jake*
NC_007799.1	205920	942218-943726	*Ehrlichia chaffeensis str. Arkansas*
NC_006831.1	302409	303748-305256	*Ehrlichia ruminantium str. Gardel*
NC_006832.1	254945	306928-308437	*Ehrlichia ruminantium str. Welgevonden*
NC_005295.2	254945	326964-328421	*Ehrlichia ruminantium str. Welgevonden*
NC_007798.1	222891	36268-37765	*Neorickettsia sennetsu str. Miyayama*
NC_009488.1	357244	1322598-1324120	*Orientia tsutsugamushi str. Boryong*
NC_010793.1	334380	379135-380647	*Orientia tsutsugamushi str. Ikeda*, complete genome
NC_009881.1	293614	864179-865686	*Rickettsia akari str. Hartford*
NC_009883.1	391896	1008161-1009668	*Rickettsia bellii OSU 85-389*
NC_007940.1	336407	537796-539303	*Rickettsia bellii RML369-C*
NC_009879.1	293613	385940-387447	*Rickettsia canadensis str. McKiel]*
NC_003103.1	272944	884601-886108	*Rickettsia conorii str. Malish 7*
NC_007109.1	315456	456383-457890	*Rickettsia felis URRWXCal2*
NC_009900.1	416276	968391-969898	*Rickettsia massiliae MTU5*
NC_000963.1	272947	772263-773769	*Rickettsia prowazekii str. Madrid E*
NC_009882.1	392021	876489-877996	*Rickettsia rickettsii str. 'Sheila Smith'*
NC_010263.1	452659	887263-888750	*Rickettsia rickettsii str. Iowa*
NC_006142.1	257363	779669-781167	*Rickettsia typhi str. Wilmington*
NC_010981.1	570417	1136001-1137446	*Wolbachia endosymbiont of Culex quin-quefasciatus Pel*, complete genome
NC_002978.6	163164	1167943-1169389	*Wolbachia endosymbiont of Drosophila melanogaster*
NC_006833.1	292805	634569-636083	*Wolbachia endosymbiont strain TRS of Brugia malayi*
NC_012416.1	66084	1289969-1291473	*Wolbachia sp. wRi *complete genome

MST distances for each cluster containing a *w*Bm gene were rounded to 2 decimal places and scaled to integers between 0 and 100. The average negative log_10 _of the e-value for the BLAST alignments between the *w*Bm gene and the other cluster members was scaled to between 0 and 1 and added to the MST integers. This resulted in a ranking score ranging from 0 to 101. The MST distances comprise the majority the score. Within-cluster e-values comprise the minority of the score, thus, for clusters with identical MST distances, the quality of alignments within each cluster determines order.

### Drug Target Similarity

The contents of the DrugBank database containing target protein sequence information was downloaded from the DrugBank website http://www.drugbank.ca/[43]. Blastp with default parameters was used to align the 805 *w*Bm protein sequences against the list of protein targets of compounds found within DrugBank. The BLAST results were filtered to remove alignments with e-values less significant than 1×10^-25^.

## Authors' contributions

AH participated in the design of the study, carried out the analyses and drafted the manuscript. PD computed minimum spanning trees and helped to draft the manuscript. JF and CC contributed to the conception of the study and helped to draft the manuscript. SK contributed to the conception of the study, and participated in its design and coordination and helped to draft the manuscript. All authors read and approved the final manuscript.

## Supplementary Material

Additional file 1 **Supplementary Table**. Contains complete MHS and GCS rankings and BLAST data for all *w*Bm genes. Click here for file
